# Trauma and the role of the wounded healer

**DOI:** 10.1192/j.eurpsy.2021.209

**Published:** 2021-08-13

**Authors:** A. Hankir, R. Zaman, F. Carrick

**Affiliations:** 1 Institute Of Psychiatry, Psychology And Neuroscience, King’s College London, London, United Kingdom; 2 Psychiatry, University of Cambridge, Cambridge, United Kingdom; 3 Neurology, University of Central Florida, Orlando, United States of America

## Abstract

**Abstract Body:**

Carl Jung used the term, ‘The Wounded Healer’ as an archetypal dynamic to describe a phenomenon which may take place in the relationship between analyst and analysand. Jung discovered the Wounded Healer archetype in relation to himself. For Jung, ‘It is our own hurt that gives the measure of our power to heal‘. Indeed, recurrent themes in the autobiographical narratives of Wounded Healers is that their experiences living with trauma inspired them to become more empathetic, driven and insightful. Many report that debilitating though the symptoms of mental illness are, the stigma is far worse. In this paper we describe the inception of an innovative anti-stigma programme, ‘The Wounded Healer’ that blends the power of storytelling and the performing arts with psychiatry and how The Wounded Healer helps to heal the wounds that were afflicted by the trauma of stigma.

**Disclosure:**

No significant relationships.

